# Second malignancies in breast cancer patients following radiotherapy: a study in Florence, Italy

**DOI:** 10.1186/bcr2860

**Published:** 2011-04-04

**Authors:** Wei Zhang, Aldo Becciolini, Annibale Biggeri, Paolo Pacini, Colin R Muirhead

**Affiliations:** 1 Epidemiology Department, Centre for Radiation, Chemical and Environmental Hazards, HPA, Chilton, Didcot, OX11 0RQ, UK; 2 Department of Clinical Physiopathology, University of Florence, viale Pieraccini 6, Florence, 50139, Italy; 3 Department of Statistics "G. Parenti", University of Florence, viale Morgagni 59, Florence, 50134, Italy; 4 Biostatistics Unit, ISPO Cancer Prevention and Research Institute, via Cosimo il vecchio 2, Florence, 50139, Italy

## Abstract

**Introduction:**

Patients diagnosed with breast cancer are often treated with surgery followed by radiation therapy. In this paper, we evaluate the effect that radiotherapy may have had on the subsequent risk of second malignancies, including the possible influences of age at treatment and menopausal status.

**Methods:**

In order to evaluate the long-term consequences of radiotherapy, a cohort study was conducted based on clinical records for 5,248 women treated for breast cancer in Florence (Italy), with continuous follow-up from 1965 to 1994. The Cox proportional hazards model for ungrouped survival data was used to estimate the relative risk for second cancer after radiotherapy.

**Results:**

This study indicated an increased relative risk of all second cancers combined following radiotherapy (1.22, 95% CI: 0.88 to 1.69). The increased relative risk appeared five or more years after radiotherapy and appeared to be highest amongst women treated after the menopause (1.61, 95% CI: 1.13 to 2.29). Increased relative risks were observed specifically for leukaemia (8.13, 95% CI: 0.96 to 69.1) and other solid cancers (1.84, 95% CI: 1.06 to 3.16), excluding contralateral breast cancer. For contralateral breast cancer, no raised relative risk was observed during the period more than five years after radiotherapy.

**Conclusions:**

The study indicated a raised risk of second malignancies associated with radiotherapy for breast cancer, particularly for women treated after the menopause.

## Introduction

Breast cancer is the most common type of cancer and the major cause of cancer-related mortality among women worldwide [1]. Many patients are diagnosed with this disease each year and are often treated with surgery followed by adjuvant radiation therapy [2]. The use of radiation therapy has increased dramatically since randomised trials in the 1980s showed equivalent outcomes for patients treated with breast conserving surgery and radiation therapy and those treated with modified radical mastectomy [3-5]. With advances in early diagnosis and treatment, breast cancer is becoming an increasingly survivable disease resulting in a large population of long-term survivors. Recent trials have shown an overall survival benefit in favour of adjuvant radiotherapy for breast cancer [6-8]. Nevertheless, there is clear evidence for the association between radiation exposure and cancer, especially from epidemiological studies of survivors of the atomic bombings in Japan [9,10], as well as from various studies of medically-exposed groups [11]. In particular, irradiation of surrounding tissues during breast radiotherapy can cause second cancers to develop within these tissues [12,13]. The second malignancy refers to a new primary cancer in a person who has survived an earlier cancer. The probability of a radiation induced second malignancy after radiotherapy is a topic that has been widely discussed [14-20]. While the benefit of radiotherapy should outweigh the risks of developing subsequent cancers, it is important to evaluate the long-term consequences of breast cancer treatment.

The aim of this cohort study was to evaluate the incidence of second primary cancers in a group of patients treated with ionising radiation therapy for breast cancer in comparison to patients not treated with ionising radiation therapy. The study involved the analysis of clinical records for female breast cancer patients treated at the University of Florence (Italy) with radiotherapy and/or chemotherapy and/or hormonal therapy from 1965 to 1994 and who were subsequently followed-up [21]. In this paper, we evaluate the effect that radiotherapy may have had on the subsequent risk of second malignancies, including the possible influence of age at treatment and menopausal status.

## Materials and methods

Data were collected on 5,248 patients with breast cancer who were submitted to radiotherapy, chemotherapy, hormonal therapy or no additional therapy at the University of Florence from June 1965 to December 1994. All of the patients had received surgery for breast cancer. A treatment schedule of 2 Gy/day, 5 days/week, for a total dose up to 60 Gy was used. Of these patients, 51.0% received radiotherapy and, for 74.0% of the patients in this group, the exposed volume was the breast only, compared with breast and lymphatic drainage in 26.0% (in 17.5% the mammary chain and supraclavicular nodes were irradiated and in 8.5% the chest wall was irradiated). The radiation source was a tele-cobalt unit (dose rate 0.80 to 1.20 Gy/minute) or linear accelerator. Different irradiation techniques with a 6 MV X-rays linear accelerator were used in later years, namely: (a) two tangential fields; or (b) two tangential fields plus direct irradiation with an electron beam of 12 MeV. Values calculated on 80 treated patients showed that, in case (a), 12% of the total dose was absorbed at a distance of 3 cm from the irradiated volume and that this value decreased progressively to 2.4% at 12 cm. In case (b), the absorbed dose was 3.8% of the total at 3 cm and 1.4% at 12 cm.

Follow-up for second malignancies was mainly conducted through direct contact with the patients at regular visits at out-patient clinics. On some visits information on health status was also verified by accompanying relatives. For this study, the cohort was assembled in 1996 and data were collected retrospectively.

Information on second malignancies was collected from hospital records and pathology reports. Vital status was assessed through demographic files at the municipality of residence of each patient. Death certificates were obtained from Mortality Registers at municipality of death for those deceased before 1981, and at the Local Health Unit thereafter. In 1984 the Tuscan Cancer Register was established; therefore, all patients' follow-up data were cross-checked with the Tuscan Cancer Registry from 1984 onwards. The end of follow-up for the subjects was chosen to be the earliest of: date of second cancer incidence, date of lost to follow-up, date of death and 31 December 1994. Only six women were lost to follow-up. The follow-up time among surviving patients ranged from a minimum of 1 year to a maximum of 30 years, with a mean of 8 years. Table 1 lists the number of patients by various therapy types and the corresponding mean age-at-treatment. The overall average age at treatment was 54.7 years. However, women who received radiotherapy only tended to be younger at the time of treatment than those who received hormonal therapy only, but older than those who received chemotherapy only.

**Table 1:** Number of women who received various types of therapy besides surgery

Type of therapy	Number of women (%)	Mean age-at-treatment (years)
No therapy	1,457 (27.8)	57.4
Radiotherapy only	1,779 (33.9)	52.7
Hormonal therapy only	663 (12.6)	62.3
Chemotherapy only	443 (8.4)	46.3
Radio- and hormonal therapy	553 (10.5)	57.8
Radio- and chemotherapy	293 (5.6)	44.3
Hormonal and chemotherapy	35 (0.7)	46.9
All three therapies	25 (0.5)	43.8
Total	5,248 (100)	54.7

The Cox proportional hazards model for ungrouped survival data [22] was used to estimate the relative risk for second cancers after radiotherapy treatment and to evaluate how the risk varied according to other factors. In particular, the hazard function at a given time was modelled as *λ(t) = λ*_*0*_*(t)*•*exp[βx]*, where *λ*_*0*_*(t) *is the baseline hazard function, *x *represents one or more explanatory variables, and *βx *is the logarithm of the relative risk. Parameter estimation and significance tests were carried out using the Epicure software [23]. In particular, Wald-type confidence intervals were computed for parameter estimates, whilst significance tests were based on χ^2 ^approximations to the distribution of the log likelihood ratio. Some patients received chemotherapy and/or hormonal therapy in both radiotherapy and non-radiotherapy groups; therefore, the relative risks of second cancers due to radiotherapy were reported both unadjusted and adjusted for chemotherapy and hormonal therapy to check if there were any confounding effects from these therapies.

The researchers carrying out the study had no identifiable details of patients forwarded to them and, therefore, ethical approval was not required under Italian laws when the project was initiated in 1996.

## Results

As shown in Table 2, among the 5,248 patients in the cohort, 261 patients (5%) developed contralateral breast cancer, 8 patients (0.15%) had leukaemia and a total of 118 patients (2.25%) developed other types of second cancers at the end of follow-up. The specifics of other types of second cancers are also shown in the table. The median time to development of second malignancy is 3 years for contralateral breast cancer, 4.5 years for leukaemia and 4.4 years for other cancers combined. Table 3 shows the estimated relative risks of all second cancers combined among patients given radiotherapy, when compared with other patients, according to the period of follow-up. Relative risks both unadjusted and adjusted for chemotherapy and hormonal therapy are presented. Also shown in this table are the numbers of cases of second cancers and the numbers of patients in the radiotherapy and non-radiotherapy groups. Second cancers that appeared within the first two years after treatment were considered to be synchronous and, hence were excluded from the analysis. Table 3 shows relative risks associated with radiotherapy for the follow-up periods of 2 to 4, 5 to 9, 10 to 14 and 15+ years respectively. The relative risk was statistically significantly less than one during the period two to four years after treatment, either with or without adjustment for chemotherapy and hormonal therapy, but was generally greater than one at longer follow-up periods. Table 4 shows that the unadjusted relative risk during the period five or more years after treatment was 1.22 (95% CI 0.88, 1.69) and was similar when adjusted for other types of therapy.

**Table 2:** Sites of second malignancies

Second cancer type	Numbers of total second cancer patients	Numbers of second cancer patients with radiotherapy
**Contralateral breast cancer**	261	103
**Leukaemia**	8	7
**Other type of cancers combined**	118	54
Lung	4	4
Liver	1	1
Kidney	6	3
Adrenal gland	1	0
Thymus	2	2
Non-Hodgkin lymphoma	4	4
Bladder	1	1
Carcinoid	1	1
Thyroid	1	0
Larynx	2	0
Sarcoma	1	0
Tongue	1	0
Myeloma	1	0
Gastrointestinal		
Colorectal	29	14
Esophagus	1	1
Gastric	18	8
Biliary tract	1	1
Anus	3	0
Gynecologic		
Cervix corpus	17	7
Cervix uteri	6	2
Uterus	1	1
Ovary	7	3
Skin		
Melanoma	6	0
Non-melanoma	1	1
Basel cell carcinoma	2	0

**Table 3:** Relative risk of all second cancers among patients given radiotherapy, by period of follow-up

Follow-up period (years)	**RR**^**a **^**unadjusted for chemotherapy and hormonal therapy (95% CI**^**b**^**)**	**RR**^**a **^**adjusted for chemotherapy and hormonal therapy (95% CI**^**b**^**)**	Case/women with radiotherapy	Cases/women with no radiotherapy
2 to 4	0.52 (0.33, 0.82)*	0.44 (0.28, 0.70)*	37/2,339 (1.6%)	53/2,377 (2.2%)
5 to 9	1.20 (0.79, 1.82)	1.08 (0.71, 1.65)	43/1,267 (3.4%)	56/1,813 (3.1%)
10 to 14	1.40 (0.75, 2.64)	1.34 (0.71, 2.52)	16/390 (4.1%)	28/972 (2.9%)
15+	0.99 (0.41, 2.41)	1.13 (0.46, 2.77)	14/230 (6.0%)	10/307 (3.3%)

* *P *<0.05.

^a ^Relative risk.

^b ^Confidence interval.

**Table 4:** Relative risk of all second cancers among patients given radiotherapy, by age-at-treatment

Age-at-treatment (years)	**RR**^**a **^**unadjusted for chemotherapy and hormonal therapy (95% CI**^**b**^**)**	**RR**^**a **^**adjusted for chemotherapy and hormonal therapy (95% CI**^**b**^**)**	Cases/women with radiotherapy	Cases/women with no radiotherapy
<50	0.96 (0.60, 1.56)	0.87 (0.54, 1.43)	32/584 (5.5%)	45/632 (7.1%)
50 to 64	1.43 (0.88, 2.31)	1.35 (0.83, 2.20)	35/549 (6.4%)	37/687 (5.4%)
65+	1.83 (0.65, 5.14)	1.69 (0.60, 4.76)	6/134 (4.5%)	12/494 (2.4%)
All	1.22 (0.88, 1.69)	1.14 (0.82, 1.58)	73/1,267 (5.8%)	94/1,813 (5.2%)

^a ^Relative risk is calculated based on a follow-up of five or more years.

^b ^Confidence interval.

In light of this pattern, we examined risks of all second cancers combined based on a follow-up of five or more years. The relative risks are calculated according to different age at the time of treatment groups (so called age-at-treatment) as shown in Table 4. Raised relative risks were observed in the 50 to 64 and 65+ year age-at-treatment groups, although none of the relative risks was statistically significantly different from one. For a cohort under follow-up, ages of patients increase as time of follow-up increases. The risk of developing a second cancer at a particular age (so-called age-at-risk) can also be calculated using the Cox proportional hazards model. Therefore we examined the relative risk by age-at-risk to examine at what age the second cancers were likely to occur, again based on a follow-up of five or more years. As shown in Table 5, raised risks were observed in the 50 to 64 and 65+ year age-at-risk groups and the relative risk in the latter group was statistically significantly greater than one (unadjusted RR 1.69, 95% CI 1.05, 2.73).

**Table 5:** Relative risk of all second cancers among patients given radiotherapy, by age-at-risk

Age-at-risk (years)	**RR**^**a **^**unadjusted for chemotherapy and hormonal therapy (95% CI**^**b**^**)**	**RR**^**a **^**adjusted for chemotherapy and hormonal therapy (95% CI**^**b**^**)**	Cases/women with radiotherapy	Cases/women with no radiotherapy
<50	0.84 (0.37, 2.02)	0.70 (0.29, 1.65)	11/569 (1.9%)	12/607 (2.0%)
50 to 64	1.14 (0.69, 1.87)	1.02 (0.62, 1.68)	28/926 (3.0%)	41/1,154 (3.6%)
65+	1.69 (1.05, 2.73)*	1.65 (1.02, 2.68)*	34/482 (7.1%)	41/1,022 (4.0%)

* *P *< 0.05.

^a ^Relative risk is calculated based on a follow-up of five or more years.

^b ^Confidence interval.

Since the 50 to 64 and 65+ year age-at-treatment groups largely or entirely consist of post-menopausal patients, we examined the relative risk of all second cancers combined in relation to menopausal status at the time of treatment. Table 6 shows the relative risk for radiotherapy separately for pre-menopausal and post-menopausal patients. Since some patients had gone through ovariectomy, they were analysed as a separate group. Table 6 indicates that patients who had undergone ovariectomy or who were post-menopausal at the time of treatment had a higher risk than did pre-menopausal patients, although only for the post-menopausal groups was the relative risk statistically significantly greater than one. A heterogeneity test showed that the relative risk differed significantly between these three groups (*P *= 0.003).

**Table 6:** Relative risk of all second cancers among patients given radiotherapy, by menopausal status

**Postmenopausal**^**a**^	**RR**^**b **^**unadjusted for chemotherapy and hormonal therapy (95% CI**^**c**^**)**	**RR**^**b **^**adjusted for chemotherapy and hormonal therapy (95% CI**^**c**^**)**	Cases/women with radiotherapy	Cases/women with no radiotherapy
Yes - natural	1.61 (1.13, 2.29)*	1.48 (1.04, 2.12)*	36/582 (6.2%)	49/1,072 (4.6%)
Yes - ovariectomy	1.40 (0.78, 2.45)	1.29 (0.73, 2.26)	4/63 (6.3%)	2/63 (3.2%)
No	0.90 (0.62, 1.32)	0.84 (0.58, 1.24)	33/621 (5.3%)	43/678 (6.3%)

* *P *<0.05.

^a ^Menopausal status at the time of treatment.

^b ^Relative risk is calculated based on a follow-up of five or more years.

^c ^Confidence interval.

Table 7 examines the relative risk for some specific types of second cancer. Leukaemia, contralateral breast cancer which refers to cancer in the opposite breast in a person with a history of breast cancer, and all other cancers combined are considered here. For leukaemia, the follow-up period was chosen to be two years or more following treatment, in view of the evidence from other studies showing a short latency period for radiation-induced leukaemia [11], whilst the follow-up period was chosen to be five years or more for other types of cancers. After adjustment for chemotherapy and hormonal therapy, the relative risk was 6.67 (95% CI 0.76, 58.00) for leukaemia and 1.70 (95% CI 0.98, 2.94) for all second cancers other than leukaemia and contralateral breast cancer combined, based on 7 and 29 cases respectively among irradiated patients. In contrast, there was no raised relative risk of contralateral breast cancer; after adjustment for chemotherapy and hormonal therapy, the relative risk was 0.82 (95% CI 0.54, 1.24), based on 41 cases among irradiated patients.

**Table 7:** Relative risk of site-specific second cancers among patients given radiotherapy

Cancer type	Follow-up	**RR**^**a **^**unadjusted for chemotherapy and hormonal therapy (95% CI**^**b**^**)**	**RR**^**a **^**adjusted for chemotherapy and hormonal therapy (95% CI**^**b**^**)**	Cases/women with radiotherapy	Cases/women with no radiotherapy
Leukaemia	> = 2 years	8.13 (0.96, 69.10)**	6.67 (0.76, 58.00)	7/2,339 (0.3%)	1/2377 (0.04%)
Contralateral breast cancer	> = 5 years	0.87 (0.58, 1.32)	0.82 (0.54, 1.24)	41/1,267 (3.2%)	67/1,813 (3.7%)
All other cancers combined	> = 5 years	1.84 (1.06, 3.16)*	1.70 (0.98, 2.94)**	29/1,267 (2.3%)	29/1,813 (1.6%)

*P <0.05; ** *P *= 0.055.

^a ^Relative risk.

^b ^Confidence interval.

Since the relative risk of second cancers other than leukaemia and contralateral breast cancer appeared to be raised after a follow-up of five or more years, we examined the relative risk by age-at-treatment. The results are shown in Table 8. Raised relative risks were observed in the 0 to 49 years and 50 to 64 years age-at-treatment groups and, in the latter instance, this relative risk was statistically significantly greater than one (RR = 2.36 (95% CI 1.08, 5.14) after adjustment for chemotherapy and hormonal therapy). In contrast, there was no evidence of a raised risk amongst those given radiotherapy at age 65 years or more. We also examined the relative risk by age-at-risk, again based on a follow-up of five or more years. As shown in Table 9, raised risks were observed in all age-at-risk groups, although only for the 65+ age-at-risk group was the relative risk close to being statistically significant greater than 1 (RR = 2.04 (95% CI 0.96, 4.35) after adjustment for chemotherapy and hormonal therapy), based on 15 cases among irradiated patients.

**Table 8:** Relative risk of other second cancers* among patients given radiation therapy, by age-at- treatment

Age-at-treatment	**RR**^**a **^**unadjusted for chemotherapy and hormonal therapy (95% CI**^**b**^**)**	**RR**^**a **^**adjusted for chemotherapy and hormonal therapy (95% CI**^**b**^**)**	Cases/women with radiotherapy	Cases/women with no radiotherapy
<50	1.54 (0.60, 3.93)	1.36 (0.53, 3.51)	9/584 (1.5%)	10/632 (1.6%)
50 to 64	2.44 (1.12, 5.30)**	2.36 (1.08, 5.14)**	18/549 (3.3%)	11/687 (1.6%)
65+	0.92 (0.18, 4.66)	0.82 (0.16, 4.13)	2/134 (1.5%)	8/494 (1.6%)

* Other second cancers are all second cancers other than contralateral breast cancer and leukaemia.

** *P *<0.05.

^a ^Relative risk is calculated based on a follow-up of five or more years.

^b ^Confidence interval.

**Table 9:** Relative risk of other second cancers* among patients given radiation therapy, by age-at- risk

Age-at-risk	**RR**^**a **^**unadjusted for chemotherapy and hormonal therapy (95% CI**^**b**^**)**	**RR**^**a **^**adjusted for chemotherapy and hormonal therapy (95% CI**^**b**^**)**	Cases/women with radiotherapy	Cases/women with no radiotherapy
<50	2.12 (0.30, 15.07)	1.64 (0.22, 12.10)	3/569 (0.5%)	2/607 (0.3%)
50 to 64	1.67 (0.71, 3.91)	1.46 (0.62, 3.43)	11/926 (1.2%)	11/1,154 (1.0%)
65+	2.07 (0.98, 4.35)**	2.04 (0.96, 4.35)	15/482 (3.1%)	16/1,022 (1.6%)

* Other second cancers are all second cancers other than contralateral breast cancer and leukaemia.

** *P *= 0.055.

^a ^Relative risk is calculated based on a follow-up of five or more years.

^b ^Confidence interval.

For comparison purposes, an analysis was conducted specifically for women who received chemotherapy or hormonal therapy only. The relative risks for a follow-up of five or more years are shown in Tables 10 and 11 respectively. The risk of all second cancers combined was not raised, either overall or for different age-at-treatment groups and in the case of chemotherapy the risk was statistically significantly less than one. However, the numbers of cases were very small.

**Table 10:** Relative risk of all second cancers among patients given chemotherapy only, by age-at-treatment

Age-at-treatment (years)	**Relative Risk**^**a **^**(95% CI**^**b**^**)**	*P*-value	Cases/women with chemotherapy	Cases/women with no chemotherapy
<50	0.37 (0.15, 0.91)	0.03	6/196 (3.1%)	34/367 (9.3%)
50 to 64	0.59 (0.17, 2.07)	0.4	3/67 (4.5%)	26/426 (6.1%)
65+	0.00	>0.5	0/11 (0.0%)	10/333 (3.0%)
All	0.42 (0.20, 0.88)	0.02	9/274 (3.3%)	70/1,126 (6.2%)

^a ^Relative risk is calculated based on a follow-up of five or more years.

^b ^Confidence interval.

**Table 11:** Relative risk of all second cancers among patients given hormonal therapy, by age-at-treatment

Age-at-treatment (years)	**Relative Risk**^**a **^**(95% CI**^**b**^**)**	*P*-value	Cases/women with hormonal therapy	Cases/women with no hormonal therapy
<50	0.57 (0.17, 1.92)	0.37	3/52 (5.8%)	34/367 (9.3%)
50 to 64	0.85 (0.38, 1.94)	>0.5	8/187 (4.3%)	26/426 (6.1%)
65+	0.52 (0.11, 2.42)	0.40	2/150 (1.3%)	10/333 (3.0%)
ALL	0.70(0.38, 1.30)	0.26	13/150 (8.7%)	70/333 (21.0%)

^a ^Relative risk is calculated based on a follow-up of five or more years.

^b ^Confidence interval.

The relative risk of all second cancers combined among patients given radiotherapy plus chemotherapy or/and hormonal therapy compared with patients given radiotherapy only is shown in Table 12. In this cohort, it appears to be that the patients who received radiotherapy along with chemotherapy and/or hormonal therapy had a lower risk of developing second cancers compared with those patients received radiotherapy alone.

**Table 12:** Relative risk of all second cancer due to combined therapy* compared with radiotherapy only

Age-at-treatment (years)	**Relative risk (95% CI**^**a**^**)**	*P-*value	Cases/women with RT plus chemotherapy or/and hormonal therapy	Cases/women with only RT
<50	0.43 (0.16, 1.14)	0.09	5/164 (3.0%)	27/420 (6.4%)
50 to 64	0.44 (0.15, 1.29)	0.14	4/142 (2.8%)	31/407 (7.6%)
65+	0.82 (0.09, 7.51)	>0.5	1/128 (0.8%)	5/106 (4.7%)
All	0.47 (0.23, 0.92)	0.03	10/334 (3.0%)	63/933 (6.7%)

* Radiotherapy plus chemotherapy or/and hormonal therapy.

^a ^Confidence interval.

## Discussion

In this study, we have used a clinical records-based cohort to analyse the effects of radiotherapy for breast cancer on the incidence of subsequent second cancers. All patients in this cohort received surgery for breast cancer. The advantage of this approach is to minimise any possible systematic difference between the irradiated and non-irradiated groups by reducing variability in the analysis. Such an approach has been used in other epidemiological studies of similar nature, as reported [17].

In our analysis, the relative risk for all second cancers appeared to be increased five or more years after radiotherapy compared to those non-irradiated. This is in agreement with the epidemiological evidence for a latency period of several years between exposure to radiation and a raised risk of second cancers [20,24-26]. The increased relative risk for all second cancers was observed in the 50 to 64 and 65+ years age-at-treatment groups (Table 4). Since these groups largely consist of post-menopausal patients, this finding may indicate an association with menopausal status. Raised relative risks were indeed observed among patients who were post-menopausal or underwent ovariectomy and, in the case of post-menopausal patients, were statistically significant. For all second cancers other than contralateral breast cancer and leukaemia, the risk variation by age-at-treatment was slightly different (Table 8). Here the increased risk was seen principally in the 50 to 64 year age-at-treatment group. Nevertheless, the raised risk was still linked with patients who had post-menopausal status or underwent ovariectomy after adjustment for chemotherapy and hormonal therapy, for example, RR = 2.16 (95% CI 1.22, 3.84) for post-menopausal patients; RR = 1.87 (95% CI 0.75, 4.66) for patients who underwent ovariectomy and RR = 1.05 (95% CI 0.52, 2.12) for pre-menopausal patients. These findings are generally in agreement with other studies that reported older age was associated with an increased risk of a non-breast second malignancy [19] and that most of the secondary lung cancers occurred more than five years after radiotherapy and in women who were >50 years at the time of their breast cancer diagnosis [18].

Increased risk of leukaemia can arise two to five years after exposure to radiation [27]. In our analysis, there was suggestion of a raised incidence of leukaemia among radiotherapy patients in the period two or more years after radiotherapy. There were seven cases in the radiotherapy group compared with only one case in non-radiotherapy group, with a relative risk of 6.67 (95% CI 0.76, 58.00) after adjustment for chemotherapy and hormonal therapy. The raised risk was not statistically significant, reflecting the small number of cases in this cohort. An increased risk of leukaemia has also been reported in previous epidemiological studies of breast cancer patients treated with radiation [28]. This raised risk might be associated with regional radiation therapy that includes an internal mammary node field, which may expose the thoracic spine to a relatively high radiation dose [13]. Due to the small number of leukaemia cases in this cohort, and there is no information available on leukaemia subtypes, analyses cannot be performed regarding leukaemia subtypes after radiotherapy in this study.

Raised risks of breast cancer have been reported in various studies of women exposed to radiation; for example, Japanese atomic bomb survivors [9,10,29], female tuberculosis patients who received multiple fluoroscopies [30,31], and female patients who received radiotherapy for various benign conditions [32]. The risk was also seen in women who had direct breast exposure prior to the age of 30 years [31-35]. However, the causes of contralateral breast cancer amongst breast cancer patients given radiotherapy are less obvious. In the Early Breast Cancer Trialists' Collaborative Group report which evaluated the effects of radiotherapy, a significantly increased risk of contralateral breast cancer was found [5]. However, in another large case-control study from Denmark, there was no significant raised risk of contralateral breast cancer among women who received radiotherapy [36]. A more recent large-scale study included 13,472 women also failed to show an increased risk of contralateral breast cancer for those received radiotherapy [37]. In some studies, it was reported that the increased risks of contralateral breast cancer were most likely observed within the first year following diagnosis of the primary breast cancer [38], or associated with patients with more advanced stage disease [39-41]. Since some patients selected for post-mastectomy radiation have a poorer prognosis than other patients, there may well be bias in estimates of the risk of contralateral breast cancer that can be attributed to radiotherapy.

In our analysis, no raised relative risk for contralateral breast cancer was observed during the period five or more years after exposure. This is in agreement with previous epidemiological studies [13,36,37,42]. Since our analyses excluded the first five years following treatment, we believe that this has excluded any effect of metastatic disease in the opposite breast. However, our result is based on a small number of cases, which makes it difficult to detect any raised risk.

Other solid cancers have also been reported to link with radiotherapy following breast cancer [14,43,44]. In our analyses, the relative risk for all second cancer excluding leukaemia and contralateral breast cancer appeared to be increased five or more years after radiotherapy. A statistically significantly increased risk was observed in the 50 to 64 year age-at-treatment group. This may indicate an association with menopausal status.

With a fairly small total number of second cancers in this cohort, the excess risk associated with radiotherapy was small over the period of follow-up. Since many of the women were still alive at the end of follow-up, the possibility of raised risks continuing several decades over radiotherapy cannot be ruled out and - based on other studies [10] - would be expected.

The study of second cancers involves special problems not inherent to most epidemiological investigations. For a second primary cancer to be classified correctly, a metastatic lesion or local recurrence of the original primary cancer must be ruled out. Other treatments may affect subsequent cancer risks for certain organs, for example, the ovary or uterus might be surgically removed. Direct comparison between the irradiated and non-irradiated groups is not without problems. The non-exposed women generally have an earlier stage disease than do women treated with radiation, and the expected number of second cancers among long term survivors is often too small, as in our analysis, to permit meaningful direct comparisons on a site-by-site basis.

It can also be argued that some of the second cancers might occur due to factors other than radiotherapy, such as a family history of breast cancer. It was estimated that up to seven percent of breast cancer cases are estimated to be due to breast cancer susceptibility genes (for example, BRCA1, BRCA2, p53 and PTEN) [45]. Although we do not have information on family history of breast cancer, a previous study [46] reported that there were increased risks of second cancers of the oesophagus, stomach, ovary, non-Hodgkin's lymphoma and leukaemia in women with a family history of breast cancer compared to those without such a history. There might also be a joint effect of radiotherapy and cigarette smoking on second cancer such as lung cancer. It was reported [43] that radiotherapy increased the risk of second primary lung cancer especially among ever smokers. Although we cannot adjust for cigarette smoking in our analysis, the effects of radiotherapy on lung cancer are evident in our cohort since all four lung cancer cases were occurred in the radiotherapy group (Table 2). This is consistent with previous studies on lung cancer risk after radiotherapy treatment [47,48]. The region of treatment may also affect the risk of the second cancer. No correlation was found with the region treated with radiation but there was a trend for a higher risk of second malignancy when the internal mammary nodes were treated [49]. Our study supports this finding since 26% of radiotherapy patients in this cohort had the mammary chain and supraclavicular nodes or chest wall irradiated.

It appears to be that the patients used radiotherapy along with chemotherapy or/and hormonal therapy had less risks of developing second cancers compared with those patients received radiotherapy alone. However, we do not have detailed information about the staging of breast cancer at the time of diagnosis and what criteria were based on for the prescription of different therapies. Therefore, it is difficult to explain the reasons why the use of chemo- and/or hormonal therapy combined with radiotherapy might lead to a lower risk of second cancer compared to the use of radiotherapy alone. However, it is interesting to note that women who only received chemotherapy or hormonal therapy did not have raised risk of second cancer, although numbers of cases may be too small to detect any raised risk in these subset analyses.

## Conclusions

This study indicated a raised risk of second malignancies associated with radiotherapy for breast cancer. The relative risk for second cancers appeared to be highest for the 50 to 65 years age-at-treatment group during the period five or more years after radiotherapy and this might be related to the patients' menopausal status. Among specific types of cancer, there were raised risks for leukaemia and for all cancers combined except leukaemia and contralateral breast cancer. The risk of contralateral breast cancer was not raised during the period five or more years after radiotherapy.

## Abbreviations

CI: confidence interval; RR: relative risk.

## Competing interests

The authors declare that they have no competing interests.

## Authors' contributions

WZ and CM participated in the data analysis and interpretation, manuscript drafting and revision. AB and AB participated in the study design, data collection, results interpretation and manuscript revision. They read and approved the final manuscript. PP was the radiotherapist involved in the project but deceased after the data collection.
